# EMERGENCY DEPARTMENT: ADULT PROTECTIVE SERVICES RELATIONSHIP IN RESPONSE TO ELDER MISTREATMENT

**DOI:** 10.1093/geroni/igac059.1595

**Published:** 2022-12-20

**Authors:** Theresa Sivers-Teixeira, Erin Thayer, Carmen Van Den Heever, Bonnie Olsen

**Affiliations:** University of Southern California, Alhambra, California, United States; University of Southern California - Keck School of Medicine, Alhambra, California, United States; University of Southern California, Alhambra, California, United States; University of Southern California, Alhambra, California, United States

## Abstract

Universal screening of older adults in emergency departments (ED) effectively improves identification of elder mistreatment (EM). To realize improved response, collaboration between ED providers and staff and local Adult Protective Services (APS) is essential. Qualitative data was derived from transcribed and coded key informant interviews with 10 APS workers and 5 ED clinical providers from 6 states. Results indicate each entity approaches EM from a different global perspective which informs key strategies to build effective response. ED providers focus on acute medical needs while APS focuses on long-term psychosocial needs. Assignment of specific individuals in both entities to be liaisons between ED and APS would support improved communication and collaboration. Education should focus on 1) capabilities and limitations in EM response and 2) clarity regarding bilateral sharing of information. Both entities report participation in multidisciplinary teams is valuable to foster collaboration on effective interventions in EM cases.

